# The “Law of Brevity” in animal communication: Sex‐specific signaling optimization is determined by call amplitude rather than duration

**DOI:** 10.1002/evl3.147

**Published:** 2019-11-21

**Authors:** Vlad Demartsev, Naomi Gordon, Adi Barocas, Einat Bar‐Ziv, Tchia Ilany, Yael Goll, Amiyaal Ilany, Eli Geffen

**Affiliations:** ^1^ Department of Biology University of Konstanz Konstanz 78464 Germany; ^2^ School of Zoology Tel Aviv University Tel Aviv 6997801 Israel; ^3^ San Diego Zoo's Institute for Conservation Research Escondido California 92027; ^4^ Wildlife Conservation Research Unit Department of Zoology University of Oxford Abingdon OX13 5QL United Kingdom; ^5^ Mitrani Department of Desert Ecology Ben‐Gurion University Midreshet Ben‐Gurion 8499000 Israel; ^6^ Rembrandt 11 Street Tel Aviv 64045 Israel; ^7^ Faculty of Life Sciences Bar‐Ilan University Ramat‐Gan 5290002 Israel

**Keywords:** Animal communication, Law of Brevity, vocal coding efficiency, vocal repertoire

## Abstract

The efficiency of informational transfer is one of the key aspects of any communication system. The informational coding economy of human languages is often demonstrated by their almost universal fit to Zipf's “Law of Brevity,” expressing negative relationship between word length and its usage frequency. Animal vocal systems, however, provided mixed results in their adherence to this relationship, potentially due to conflicting evolutionary pressures related to differences in signaling range and communicational needs. To examine this potential parallel between human and animal vocal communication, and also to explore how divergent, sex‐specific, communicational settings affect signaling efficiency within a species, we examined the complete vocal repertoire of rock hyraxes (*Procavia capensis*). As male and female hyraxes differ in their sociality levels and male hyraxes vocal repertoire is dominated by sexual advertisement songs, we hypothesized that sex‐specific vocal repertoires could be subjected to different signaling optimization pressures. Our results show that the sexes differ in repertoire size, call usage, and adherence to coding efficiency principles. Interestingly, the classic call length/call usage relationship is not consistently found in rock hyraxes. Rather, a negative relationship between call amplitude and call usage is found, suggesting that the efficiency of the vocal repertoire is driven by call amplitude rather than duration. We hypothesize that, in contrast to human speech that is mainly intended for short distance, the need for frequent long‐range signaling shapes an animal's vocal repertoire efficiency according to the cost of call amplitude rather than call length. However, call duration may be a secondary factor affecting signaling efficiency, in cases where amplitude is under specific selection pressures, such as sexual selection.

**Impact Summary**

Searching for the origins of human language, we often look for similarities with animal communication. Beyond the analogous sound producing mechanisms, there are statistical principles related to production and communicational efficiency. One is the Law of Brevity, claiming an inverse relationship between word length and usage that optimizes informational coding against the effort of sound production. Despite being a fundamental principle in human language, its applicability to animals is controversial. The potential lack of fit of animal vocal communication systems to Law of Brevity might be a result of different pressures shaping human and animal vocal repertoires and also stem from the differences between male and female vocal repertoires, which is frequently the case in animals. Here, we examined sex‐specific differences in the vocal repertoire and communicative efficiency of the rock hyrax (*Procavia capensis*), a mammal whose social structure is that of social females and mostly solitary bachelor males. We found sex‐specific differences in the hyrax vocal repertoire. The social females have a larger vocal repertoire with more affiliative call types than the males. The bachelor male repertoire, which includes male‐specific advertisement songs, demonstrates a better fit to linguistic efficiency laws. Interestingly, the hyrax vocal repertoire does not comply with the classic linguistic call lengths and usage patterns, instead featuring a negative relationship between call amplitude and usage, suggesting amplitude as the dominant factor in shaping hyrax communicative efficiency. Those differences might be related to signaling distance, as human language is mostly intended for short‐range communication, whereas long‐range, high‐amplitude calling is frequent in animals. Thus the differences between the communication requirements of humans and animals might have resulted in the informational coding efficiency being driven by different evolutionary forces, respectively.

## Background

Despite the fact that animal calls fail to reach the complexity and production flexibility of human language, there are numerous anatomical (Fitch and Reby 2001; Fitch and Suthers 2016; Fitch 2018) and neural analogies (Jurgens 2009; Fitch and Suthers 2016; Fitch 2018) as well as fundamental communication principles shared between human and animal communicational systems (Marler 1998; Pika et al. 2018). One of the basic aspects of any communicational system is the efficiency of its informational transfer. A potential set of metrics enabling this efficiency assessment is that of Zipf's empirical “laws,” formulated to assess the efficiency of informational coding in languages (Zipf 1945, 1949). One of those laws is the “Law of Brevity” (Zipf 1999), according to which the length of words is negatively correlated with the frequency of their use (Ramon et al. 2013). A related “principle of least effort” (Hailman et al. 1985) predicts an approximately monotonic relationship between word probability of occurrence and its rank of use (the frequency of a word decays as a power law of its rank; Cancho and Solé 2003). The reasoning behind these relationships is related to the economic compromise between information transfer and the articulatory effort associated with the production of sound (Hailman et al. 1985; Manning and Schutze 1999; Cancho and Solé 2003). This is well demonstrated in the process of language evolution, as longer words are often shortened by speakers when in frequent use (Hernández‐Fernández et al. 2016; e.g., the word “television” has been abbreviated to TV).

Zipf's laws have been previously applied in assessing the economy of animal communication (Dawkins 1976; Hailman et al. 1985), but with somewhat contrasting results. In birds, black‐capped chickadee (*Parus atricapillus*) calls follow the least effort rule but not the Law of Brevity (Hailman et al. 1985). The least effort rule was also shown in the spectacled warbler (*Sylvia conspicillata*; Palmero et al. 2014) and the skylark (*Alauda arvensis*) songs (Briefer et al. 2010). In primates, the vocalizations of the Formosan macaque (*Macaca cyclopis*; Semple et al. 2010) and the chimpanzee's (*Pan troglodytes*) gestural communication follow Zipf's principles of informational compression (Heesen et al. 2019). In contrast, the common marmoset (*Callithrix jacchus*) and the golden‐backed uakari (*Cacajao melanocephalus*) vocal repertoires do not follow the predicted call duration/usage frequency pattern (Bezerra et al. 2010). (Note: The latter results have been criticized for insufficient sampling and for the inclusion of long‐distance calls, for which the pressure for brevity is reduced; Ferrer‐i‐Cancho and Hernández‐Fernández 2012.)

The species‐specific adherence to Zipf's laws could be related to differences in social environments, which impose different communication constraints on the signaling systems. In line with the social complexity hypothesis, the diversity and frequency of communication are dependent on the social settings (Freeberg et al. 2012). As has been shown in comparative studies assessing phylogenetically close species, frequently interacting individuals require a more elaborate (Freeberg and Lucas 2012; Pollard and Blumstein 2012; Bouchet et al. 2013) and perhaps also more efficient communication system. Similar pressures might function within a species, for example, in cases of sex‐specific divergence in levels of sociality (Wittemyer and Getz 2007; Demartsev et al. 2016b) or due to asymmetry in sexually selected signals. Signals and even whole signaling repertoires often demonstrate exaggerated sexual differences implying adaptation to different communication environments. Zipf's laws offer a tool for comparing communication efficiency both across (McCowan et al. 1999, 2005) and within species. Focusing on sex‐specific adherence can isolate effects driven specifically by communicational needs, while controlling for differences related to vocal learning abilities (Janik and Slater 2000), habitat, communication modality, and cognition‐driven communication complexity (Pika et al. 2003; Pika and Mitani 2006). It has the potential for showing how sex‐specific communication needs affect the extent a communication system fits to the optimal statistical signaling efficiency.

Zipf's laws have previously been tested only in avian and primate taxa (Bezerra et al. 2010; Briefer et al. 2010; Hernández‐Fernández et al. 2016). To broaden the assessment of the phylogenetic spread and the general relevance of this linguistic theory to animal vocal communication, we focus here on a distant mammalian species, the rock hyrax (*Procavia capensis*). This is a social mammal that employs the acoustic modality as its main channel of communication (Koren and Geffen 2009, 2011) and has an extensive and well‐described acoustic repertoire (Fourie 1977). Rock hyraxes live in mixed‐sex groups consisting typically of one adult resident male and up to 20 females with their offspring (Koren et al. 2006; Ilany et al. 2011). Upon reaching sexual maturity, the young males usually disperse to become bachelors, whereas the females mostly remain in their natal groups (Hoeck 1989) and maintain stable social bonds (Barocas et al. 2011). Although bachelor males often remain in the periphery of colonies (Koren et al. 2008), their social interactions are limited to mostly aggressive encounters with other males (Barocas et al. 2011) and seasonal courtship with the group females (Bar Ziv et al. 2016). Thus, hyraxes exhibit a high variation in their sociality levels, with females and resident males being more social than bachelors. These differences have allowed us to examine the consistency of the Law of Brevity and its dependence on sex‐specific and potentially socially determined communication requirements.

We hypothesized that in agreement with the Law of Brevity, which favors short signals, an inverse relationship would be found between signal duration and signal usage in the highly social hyrax females. As long‐range acoustic transmission favors longer signals that are more robust to interference, this might, create a stabilizing pressure against the Law of Brevity (Ferrer‐i‐Cancho and Hernández‐Fernández 2012). We therefore predicted that the negative relationship between signal duration occurrence and signal usage would be less evident in the male repertoire, due to their potentially limited short‐range communication requirements. Following previously expressed reservations (Ferrer‐i‐Cancho and Hernández‐Fernández 2012) that long‐range, high‐amplitude calls (Bradbury and Vehrencamp 2011) shift the balance of cost for call production, we examined whether the exclusion of long‐range calls might reveal a better fit of the sub‐repertoire to the predicted duration/usage pattern. We hypothesized that loud long‐range calls, such as alarm trills and male advertisement songs, would not comply with the Law of Brevity. The energetic cost of a vocal signal is mainly determined by a weighted summation of its amplitude, duration, and rate (Holt et al. 2015). Thus, we hypothesized that a combination of these parameters might serve as a better representation of the cost of call production rather than a narrow focus on call duration or call amplitude independently and consequently reveal a negative relationship between the production cost of calls and their usage.

## Methods

### ETHICAL STATEMENT

The study was conducted under permits from the Israel Nature and Parks Authority for capturing, handling, and sampling hyraxes at the Ein Gedi Nature Reserve (2005/17687, 2007/27210, 2008/31138, 2009/32871, 2010/37520, 2011/38061, 2012/38400, 2013/38803). No long‐term stress or interference effects were detected. The social groups and the overall population in the research area remained stable throughout the study period.

### FIELD PROTOCOL

The study was conducted at the Ein Gedi Nature Reserve, Israel (31^o^28′N, 35^o^24′E), where five mixed‐sex hyrax groups and numerous bachelor males have been continuously monitored since 1999. Field procedures followed previously published protocols (Koren et al. 2006; Koren et al. 2008; Barocas et al. 2011; Demartsev et al. 2014). Wild rock hyraxes were trapped using live box traps (Tomahawk Live Trap Co, USA) baited with cabbage and kohlrabi. Trapped animals were anaesthetized by intramuscular injection of ketamine hydrochloride (10 mg/kg). Each hyrax was individually marked with a subcutaneous transponder (DataMars SA) and either an ear tag (approx. 0.25 grams per tag) or a light numbered collar (approx. 5 g). Following anesthesia recovery (at least 120 min), the animals were released back at their capture sites and resumed full normal activity.

### DATA COLLECTION

Between 2009 and 2013, 10 bachelor males (mean age 5.2 ± 1.4 SD) and nine adult female hyraxes (mean age 6.2 ± 2.2 SD) were fitted with collar‐mounted, miniature audio recorders (Edic‐mini Tiny B22 or Edic‐mini Tiny 16 B25, Telesystems LTD, Russia). Each individual was recorded once for a consecutive period of up to 14 days. The recorder was set to a sampling frequency of 22,050 Hz and the frequency band was 100–10,000 Hz. A voice activation system was set to start operating at a sound amplitude higher than 37 dB SPL, a threshold previously found to allow recording of all vocals, while ignoring ambient noise (Ilany et al. 2013). Following the onset of the recording, it would continue for 10 s after the last sound heard above this threshold level. The date and time of each recording were saved with the file.

All the recorders were fitted between April and August of each year and all the recorded individuals originated from the same research site (approximate area of 2 km^2^). The individuals were chosen based on their chances of being re‐captured for recorder removal (having a previous history of at least one re‐capture per week of trapping). These recording units provide an individual's full vocal repertoire (including low‐amplitude, short‐range calls) and register a realistic call usage over an extensive period. A total of 149,671 calls, comprising 476 min of vocal recording, were collected and processed, with an average of 7127 ± 4790 (SD) calls per individual (24.9 min ± 14.4 SD net vocalization time). The average recording period for each individual in the field was 5.2 days ± 1.4 (SD).

We sampled call usage by counting the occurrences of each call type, as performed in previous work (Ferrer‐i‐Cancho and Lusseau 2009; Bezerra et al. 2010; Ferrer‐i‐Cancho and Hernández‐Fernández 2012; Luo et al. 2013; Hernández‐Fernández et al. 2016). Furthermore, the standardized, on‐source audio recording enabled us to reliably measure call amplitude, avoiding the effect of distance from the caller (Wyman et al. 2008).

### ACOUSTIC ANALYSIS

The audio files were analyzed using Avisoft SAS LabPro software, version 5.2.07 (Avisoft Bioacoustics, Germany). Spectrograms were generated for each vocal file, measured at 512 FFT length, 100% frame, using a Hamming window, and the acoustic units were identified and categorized manually by human visual‐auditory inspection. In cases where more than one individual was recorded simultaneously, the focal calls were distinguished from nonfocal calls by comparing their relative amplitudes. As the automatic gain correction function of the recorder was deactivated and the microphone was positioned just below the animals’ head, close‐fitting to the laryngeal area, we expected a minimal number of cases in which nonfocal calls might be mistakenly assigned as focal.

**Figure 1:**
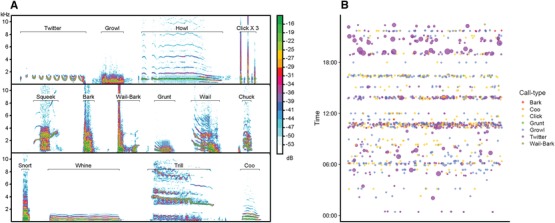
(A) Sample spectrograms of typical exemplar for each of the identified call types in rock hyrax vocal repertoire. (Twitter, Growl, Squeek Bark, Wail‐Bark, Grunt, Wail, Snort, Whine, and Coo—according to Fourie 1977; Howl, Click, Chuck, and Trill—newly defined in this study.) (B) Relative call usage frequency of a single adult female hyrax over 24 h. Color codes denote respective call type. Point size represents the cumulative duration of the respective call type. Points are jittered because of overlaps.

Acoustic communication in rock hyraxes includes functional call types for various contexts (e.g., alarm, aggression, appeasement, quality advertisement, distress, excitement, food competition, and more; Fourie 1977). Fourie's (1977) call classification of captive rock hyraxes was used as a reference. Due to the poor quality of the spectrogram samples in the original paper, we could not fit all the recorded calls to this predetermined repertoire, and nor could we detect all the call types in our recordings. Out of 18 acoustic call types classified by Fourie for adult hyraxes, we could reliably identify 10 types. Four new call types were defined and named by us (Fig. 1A, Supporting Information Data 001–014). Thus, our final call classification comprised both previously defined calls and new call types identified and named in this study. Each call type represented a distinguishable acoustic unit (Anikin et al. 2018). The definition and identification of call types was performed by four observers and a consensus was reached regarding any ambiguities.

All recorded calls were manually marked on the spectrogram using the Avisoft SASLabPro cursors. A blind verification of the call types was performed by two independent observers. Each received ∼20 random exemplars from each call type as a collection of anonymized audio files, together with approximately five named samples for each call type. Each observer independently reported the identification for the anonymized recordings. The original call classification and the verification results were tested for interobserver agreement by calculating Fleiss’ kappa coefficient (Gwet 2008). The rater agreement *ƙ* = 0.725 (*n* = 259, raters = 3) indicated substantial agreement. Calculations were done in R, version 3.5.2 (package irr version 0.841).

The mean duration and mean amplitude of each call type per individual were calculated from the measurements extracted by SASLabPro's automatic parameter measurement function. Subsequently, the number of occurrences for each call type was summarized.

*Call occurrence frequency* (the number of times a call type appeared out of the total calls within an individual repertoire) was used as an estimate of call type usage.

Although both males and females have extensive acoustic repertories, it is almost exclusively adult males that produce songs (Koren et al. 2008) that function as a quality advertisement signal (Koren and Geffen 2009; Demartsev et al. 2014). Male songs presented a conceptual challenge in determining the relevant classification unit. The songs are composed of three distinguishable vocal elements that are arranged in up to 90 singing bouts, separated by a silent interval of ∼1 s (Demartsev et al. 2014). Previous analyses have considered songs both as single units (Ilany et al. 2013; Demartsev et al. 2017) and as sequences of the specific elements that comprise them (Koren and Geffen 2009, 2011; Demartsev et al. 2016a). Both approaches have their shortcomings. The single unit approach ignores the fact that, with respect to duration, hyrax song is not comparable with an individual call, lacks the continuity of sound production, and the different vocal elements might bear different informational content (Koren and Geffen 2009). However, as songs have a stereotypic progression pattern with a typical beginning and end, they might contain synergetic information that could be lost by treating then only in terms of their elements (Demartsev et al. 2017). We do not know the receivers’ perception unit (Kershenbaum et al. 2016), and various element combinations in the song might have communicative significance (Demartsev et al. 2017). Therefore, in the current study we performed two analyses: once with each song treated as a single call type, and once with songs treated in terms of their separate elements.

### STATISTICAL ANALYSIS

From the total list of call duration and call amplitude measurements, a series of individual call type–based averages were calculated. The dataset used for statistical analysis is openly available at https://doi.org/10.17632/yzw5bjhcwb.1. For each individual animal, there is one average call duration and one average call amplitude value per each call type. These series of individual averages were used as predictors in our statistical analysis. We employed this approach to address potential biases related to individual differences in sample size and pseudo‐replication.

In an attempt to incorporate both call duration and amplitude into a single parameter reflecting general call production effort (CPE), we used the product of call duration and call amplitude to calculate a single measure of mean CPE for each specific call type and examined its effect on occurrence frequency. To address the bias created by the variable with the larger values in the product, we standardized (i.e., scaled between 0 and 1) both call duration and call amplitude prior to multiplication.

We used a Generalized Estimation Equation (GEE) mixed model to test for trend differences between the sexes in the relationship between call type occurrence frequency and three predictors (call type duration, call type amplitude, and CPE). The effects of call type duration and call type amplitude were evaluated using a single model, whereas the effect of CPE was evaluated separately. In our models (Table 1), we added the interaction between sex and the focal predictor, and set individual identity as a random effect. The same analysis was performed twice: once in which songs were treated as a single call type (i.e., we calculated the duration of the whole song as a function of its frequency), and once in which songs were separated into their individual elements (i.e., we calculated the mean duration of each element within the song separately as a function of its frequency). In all the models, we used the gamma distribution (lowest AICc = –466.5) and a log as the link function (due to the expected exponential decay in frequency with the increase in call type duration or amplitude).

**Table 1:** The model effect of each of the two regressors (mean call duration and mean call amplitude) on call occurrence frequency in hyrax

Model term	Estimate (SE)	*χ* ^2^_1_	*P*‐value
*Song treated as a single call type (n = 151)*			
Sex	−0.951 (0.443)	4.6	**0.032**
Element duration	−0.105 (0.021)	5.6	**0.018**
Element amplitude	−0.062 (0.015)	54.1	**<0.001**
Sex × Element duration	1.772 (0.660)	7.2	**0.007**
Sex × Element amplitude	−0.015 (0.019)	0.6	0.438
*Song split into separate elements (n = 170)*			
Sex	0.703 (0.457)	2.4	0.124
Element duration	−0.776 (0.256)	1.6	0.208
Element amplitude	0.005 (0.014)	15.2	**<0.001**
Sex × Element duration	2.444 (0.707)	11.9	**0.001**
Sex × Element amplitude	−0.082 (0.018)	19.7	**<0.001**
*Long‐range calls excluded (n = 130)*			
Sex	−1.349 (0.458)	8.7	**0.003**
Element duration	−0.235 (0.223)	21.9	**<0.001**
Element amplitude	−0.053 (0.015)	48.5	**<0.001**
Sex × Element duration	4.396 (0.840)	27.4	**<0.001**
Sex × Element amplitude	−0.028 (0.019)	2.2	0.141

The Wald *χ* ^2^ test evaluated the significance of the effects. *df* = 1 in all cases. Each column shows the model estimate (slope) for the respective covariate with call occurrence frequency. Models were fitted using the gamma distribution and a log link function. Significant *P*‐values are in bold. *n* is the sample size. The sex reference is female.

Additionally, a similar GEE model was fitted to the subset of the data that did not include long‐range calls of males and females (songs and alarm trills). This test was added to control for the possibility that loud, long‐range calls, being subjected to specific pressures related to signal transmission over large distances, might mask the adherence of the rest of the repertoire to the duration/frequency rule (Table 1).

The correlation between mean call duration and mean call amplitude was very low (*r* = –0.097). In contrast, the correlation between CPE and mean call duration, and CPE and mean call amplitude was substantially higher (*r* = 0.8601 and 0.339, respectively). Thus, we tested for CPE effects in a separate model.

## Results

### GENERAL VOCAL PRODUCTION

**Figure 2:**
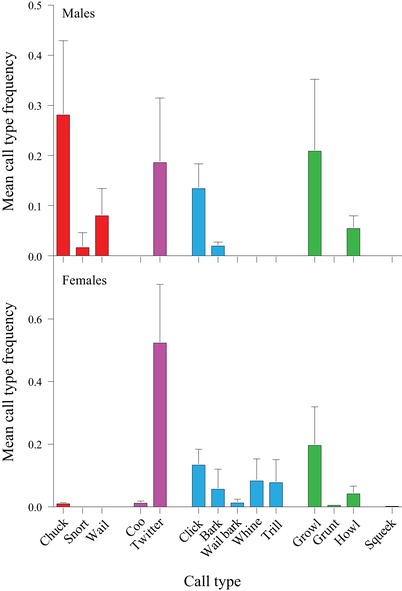
Mean frequency (±SD) of the various call types in male and female rock hyraxes. The behavioral contexts of each call type are denoted by color (red—song elements; purple—affiliative behavior and appeasement; blue—fear, arousal, and alertness; green—agonistic and threat behavior; black—unknown function).

Fourteen call types were identified and classified from the collected recordings (Fig. 1A), and individual call‐usage frequencies were summarized over the course of the recorded time (Fig. 1B). There was a difference between male and female vocal repertoires, with females producing 12 call types and males producing only eight (Fig. 2). Males and females shared six call types—two call types were uniquely male and six call types were uniquely female. We did not find a significant difference in net vocalization times (without silent intervals) between males and females, with females calling for a median of 4.8 min (Interquartile Range [IQR] = 13.5) per day and males calling for a median of 6.4 min (IQR = 5.2) per day (Permutation test on mean difference, *n* = 18, 1000 permutations, *P* = 0.201). The occurrence frequencies of shared call types differed between males and females. Notably, the frequency of the Twitter type was ∼18% in males and ∼52% in females (Fig. 2). The most frequent call type in males was Chuck, a song component with 28% occurrence, whereas in females Chuck occupied ∼1% of their vocal production (Fig. 2).

### CALL METRICS AND CALL OCCURRENCE FREQUENCY

Our results showed a between sex difference in most call occurrence frequency trends, as reflected by the significant interactions between sex and the predictors (Table 1).

**Figure 3:**
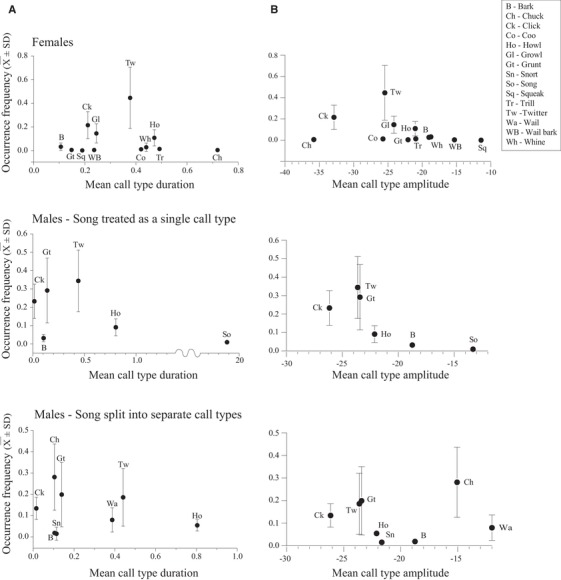
Sex‐specific call‐type usage as a function of (A) call duration (s) and (B) call amplitude (dBFS) in rock hyraxes. (Dots denote mean values with SD error bars.) In several cases SD bar range is too small to be displayed. Call usage is estimated as “Occurrence frequency”—proportion of specific call count out of total number of emitted calls.

The female vocal repertoire did not comply with the law of brevity. The model revealed a significant positive association (long call types occurred more frequently than short ones; estimate [SE] = 1.67 [0.66], *χ* ^2^_(1)_ = 6.3, *P* = 0.011; Fig. 3A) between call type duration and call type occurrence frequency (the number of times a call type appeared out of the total number of calls produced by the individual). This relationship might be driven by the Twitter, which is the most frequently used call type and also one of the longest.

For the male vocal repertoire, as we do not know the relevant perception unit of the “song” call type, all analyses were performed twice: once with each song treated as one vocal unit, and once with songs treated in terms of their separate parts (see Methods for more details). The male vocal repertoire demonstrated a relationship that fitted to the Law of Brevity. Shorter call types were more frequently used with regard to both separate song elements (estimate [SE] = –0.78 [0.26], *χ* ^2^_(1)_ = 9.2, *P* = 0.002) and complete songs (estimate [SE] = –0.11 [0.02], *χ* ^2^_(1)_ = 25.7, *P* < 0.001; Fig. 3A).

When the effects of call amplitude on the occurrence frequency were considered, females demonstrated a negative relationship (estimate [SE] = –0.08 [0.02], *χ* ^2^_(1)_ = 44.9, *P* < 0.001; Fig. 3B), implying that frequently used calls in female rock hyraxes are quieter. In males, similar results were found, but only when songs were treated as a single call type (estimate [SE] = –0.06 [0.02], *χ* ^2^_(1)_ = 17.1, *P* < 0.001; Table 1).

Last, we did not find a significant relationship in females between CPE (CPE = call duration × call amplitude) and call occurrence (estimate [SE] = –1.81 [1.14], *χ* ^2^_(1)_ = 2.5, *P* = 0.113; Table 2). In males, the relationship between occurrence frequency and CPE was negative both when they were considered as a whole (estimate [SE] = –4.81 [0.65], *χ* ^2^_(1)_ = 54.0, *P* < 0.001) and when songs were considered as a sequence of individual elements (estimate [SE] = –1.61 [0.46], *χ* ^2^_(1)_ = 12.3, *P* < 0.001), meaning that calls requiring less effort to produce were more frequent.

**Table 2:** The model effect of mean call production effort (CPE) on call occurrence frequency in hyrax

Model term	Estimate (SE)	*χ* ^2^_1_	*P*‐value
*Song treated as a single call‐type (n = 151)*			
Sex	−0.440 (0.130)	11.4	**0.001**
CPE	−4.810 (0.655)	3.1	0.078
Sex × CPE	−37.732 (26.877)	2.0	0.160
*Song split into separate elements (n = 170)*			
Sex	−0.175 (0.146)	1.4	0.231
CPE	−1.605 (0.458)	7.7	**0.006**
Sex × CPE	−0.204 (1.231)	0.0	0.868
*Long‐range calls excluded (n = 130)*			
Sex	−0.748 (0.198)	14.2	**<0.001**
CPE	−1.139 (0.486)	0.3	0.604
Sex × CPE	1.416 (1.660)	0.7	0.394

The Wald *χ* ^2^ test evaluated the significance of the effects. *df* = 1 in all cases. Each column shows the model estimate (slope) for the respective covariate with call occurrence frequency. Models were fitted using the gamma distribution and a log link function. Significant *P*‐values are in bold. *n* = sample size. The sex reference is female.

### LONG‐RANGE CALL EXCLUSION

We repeated the analysis, testing the effect of call type duration and amplitude on call type usage after excluding long‐range calls (alarm trills in females and songs in males). In females, the results were similar to those obtained from the full vocal repertoire (Table 1). The relationship between call duration and occurrence frequency remained positive (estimate [SE] = 4.16 [0.81], *χ* ^2^_(1)_ = 26.4, *P* < 0.001). The relationship between call amplitude and call occurrence frequency remained negative (estimate [SE] = –0.08 [0.01], *χ* ^2^_(1)_ = 45.6, *P* < 0.001). The relationship between CPE and occurrence frequency was insignificant (estimate [SE] = 0.28 [1.58], *χ* ^2^_(1)_ = 0.0, *P* = 0.861; Table 2). In males, the relationship between call amplitude and call occurrence frequency (estimate [SE] = –0.05 [0.02], *χ* ^2^_(1)_ = 12.4, *P* < 0.001) and between CPE and occurrence frequency (estimate [SE] = –1.14 [0.48], *χ* ^2^_(1)_ = 5.5, *P* = 0.019) remained negative, but the relationship between call duration and occurrence frequency (estimate [SE] = –0.24 [0.22], *χ* ^2^_(1)_ = 1.1, *P* = 0.293) was not significant. See Table 3 for a pairwise summary.

**Table 3:** A qualitative summary of rock hyrax sex‐specific repertoire adherence to call duration, amplitude, and call production effort (CPE) based Zipfian efficiency

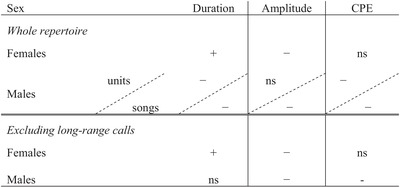

The table summarizes the trends in males and females detected by the GEE model. “−”denotes significant negative relationship between call usage and the tested measure (e.g., shorter calls are more frequently used); “**+**” denotes significant positive relationship between call usage and the tested measure (e.g., louder calls are more frequently used); “ns” denote no significant relationship. Male whole repertoire was analyzed twice: “song”: each song treated as one vocal unit; and “units”: with songs treated in terms of their separate parts.

## Discussion

Our results demonstrate a clear distinction between male and female hyrax vocal repertoires, with females producing more call types and using the shared call types in different proportions to those of males (Fig. 2). This distinction could potentially be explained by the differences in social setting between the group‐living females and the mostly solitary bachelors. The level of communication between frequently interacting conspecifics necessitates for a more refined signaling system (Freeberg et al. 2012). It has been suggested that the higher vocal variability in red‐capped mangabey (*Cercocebus torquatus*) females is related to their stronger social integration in comparison to males (Bouchet et al. 2010). In our case, hyrax females employed a larger vocal repertoire, with more tonal, high‐frequency, and potentially affiliative call types (Fig. 1A, Coo, Whine, and Squeak) that do not feature in the male repertoire.

Concerning the Law of Brevity, hyrax females not only did not comply with it but, rather, they demonstrated an opposite relationship, with the longest call types being the more frequently used (Table 3; Fig. 3A). Males, in contrast, did show a fit to the brevity pattern, with shorter calls being more frequently used. When long‐range calls were omitted from the analysis, as suggested by Ferrer‐i‐Cancho and Hernández‐Fernández (2012), females still presented a significant opposite relationship to that predicted by the Law of Brevity, whereas male results became not statistically significant (Table 1). Thus, the relationship between call type occurrence and duration is inconsistent between the sexes and in general, our results do not support the notion of shorter call types appearing more frequently in the hyrax vocal repertoire.

In contrast, the relationship between call‐type occurrence and amplitude did follow the predicted pattern, with louder calls being less frequent in both sexes (Table 3, Fig. 3B). From a communication perspective, louder calls are more likely to be used for long‐distance communication and/or in high‐urgency situations (Claudia and Kurt 2002). Thus, their occurrence frequency reliably reflects their rare usage (Gustison and Townsend 2015), in comparison to the quiet, short‐range contact calls that continuously mediate social interactions among conspecifics. However, given the social differences between bachelor males and group‐living females, we expected that this relationship would be weaker in the mostly solitary bachelor males. When male songs were analyzed as separate elements (and not as a single unit), the pattern of louder calls being less frequent was lost. Hyrax songs are high‐amplitude, long‐range signals, and each song can be composed of hundreds of vocal elements. Examining the separate elements for their occurrence frequency potentially offers a better representation of male repertoire call‐usage/amplitude proportions and a better reflection of the effort involved in producing numerous high‐amplitude calls, in contrast to each song being considered as one unit. When the loudest long‐range calls are omitted, the relationship between call‐type occurrence and amplitude in both sexes becomes similarly negative (Table 3).

To examine a communication system's compliance with Zipf's Law of Brevity, an extensive sampling of communication units is required. Tests of language compliance with the Law of Brevity have used large corpuses of text or extensive collections of spoken language (Ferrer‐i‐Cancho and Hernández‐Fernández 2012). Our work here was based on an automated collection of on‐source audio recordings, delivering a realistic, amplitude‐unbiased sample of the subjects’ vocal production, unavailable in previous animal studies. As full compliance with the Law of Brevity could not be shown, despite our exhaustive sampling of the hyrax vocal repertoire, our findings support the idea that a rigid dependency of signal duration on signal frequency is too simplistic (Semple et al. 2010). Bezerra et al. (2010) suggested that although brevity can be favored by natural selection, there are additional forces acting on signal duration that affect the costs of signaling. Amplitude could be one such force, especially for urgent signals that need to be communicated briefly and clearly. Increased amplitude in these signals might shift the balance, making them costlier than the longer and low‐amplitude signals used for close‐range communication. Alternatively, the relationship between amplitude and usage might be simply determined by call context and function. The infrequency of high‐amplitude calls is associated with the occurrence of uncommon events such as agonistic interactions and distress, rather than based on coding and energetic efficiency.

Another candidate metric by which to assess the cost of signal production is that of the CPE. In male hyraxes, this seems to fit the general “least effort” paradigm (Zipf 1949) of the most frequently used call types requiring the least production effort. For females, however, this metric is not significant, possibly due to the contradicting effects of call duration and call amplitude. There is a lack of knowledge regarding the relative energetic costs of call duration and amplitude in vocal production. In this work, we assumed both parameters to have equal contribution to CPE, although it is likely that one might be costlier than the other (Oberweger and Goller 2001; Noren et al. 2013). Nevertheless, the CPE results stress out that the main source of difference between male and female repertoire optimization is likely to be due to the different effort‐determining factor. Male repertoire optimization is likely to be affected by songs, which are the longest and loudest calls in the male repertoire, occupying 40.1% of the total vocal time per day and 78.1% of the total energy invested in vocalization per day (Ilany et al. 2013). The songs’ function, as a long‐range advertisement, requires them to be high amplitude, and this might outweigh the pressures for reduction of high‐amplitude signals. It is possible that as a means for balancing the costs of amplitude, songs are subjected to optimization pressure on call duration, as the next effort‐determining factor. This results in a different efficiency equilibrium for male and female vocal repertoires. Omission of long‐range calls does not affect our findings for females, potentially indicating an even dispersion of the female repertoire over transmission distances and homogeneity of optimization pressures. The male repertoire, however, is constrained by the unique requirements of advertising songs and fails to reach amplitude‐based optimization with their inclusion (analyzed at the level of separate units). If male songs are omitted, male routine social communication calls show a fit to amplitude‐based optimization, similar to that of the female repertoire and no longer show duration‐based optimization, as with their full repertoire. This suggests that call amplitude is the main factor affecting call type usage in rock hyraxes, whereas duration is a factor mainly in male songs, which are a special case in the repertoire. However, at this point neither energetic efficiency nor contextual usage frequency can be ruled out as driving forces behind those relationships.

In terms of the relevance of linguistic statistic universals to animal vocal communication, the general paradigm of “least‐effort” should be conceptually valid, as the majority of previously tested animal species, including rock hyraxes, have demonstrated at least partial compliance with it. Perhaps it is the application of “word length,” as the efficiency‐determining metric for animal calls, which should be reconsidered. Animal vocal signals and human speech are subject to different selective pressures. The Law of Brevity considers a “word” to be the basic linguistic unit, but an animal call type is not necessary parallel to a human word (Luo et al. 2013). Furthermore, certain animal call types are dispersed on a graded scale (Keenan et al. 2013), with variation in duration reflecting levels of arousal or urgency (Wilson and Evans 2012). A predominance of graded calls in a species’ vocal repertoire can make the statistical characteristics of brevity irrelevant, as the same call type can occupy a wide temporal range.

Further, animals often communicate in acoustic environments and mediums that greatly differ from those of humans. Human speech seems to be adapted for short‐range transmission (Naguib and Wiley 2001). “Shouted speech” is less intelligible to human listeners (Pickett 1956), and the comfortable distance for a social conversation is up to 3.5 meters (Scott 1984). In contrast, animals frequently contend with variable transmission distances and manage to routinely transmit information over many kilometers (Harrington and Mech 1979; Zuberbuehler et al. 1997). Adaptations for long‐ vs. short‐range transmission are driven by opposite pressures. Long‐range transmission relies on sound power, which often impairs sound modulation complexity and decreases the informational content (Titze and Palaparthi 2018). This might provide an explanation for why the word length/usage relationship is considered universal in human communication, whereas animal communication does not consistently comply with it. The probable pressure for the informational complexity of human vocal communication might have limited the use of high‐amplitude vocalizations. In parallel, the invention of artificial means of long‐range communication (e.g., drumming [Seifart et al. 2018], smoke signals [Turpin 1984]) could have replaced the need for long‐range vocal transmission. As a pure speculation at this stage, the elimination of the amplitude constraint might have allowed human languages to reach a signal duration/signal occurrence optimization (brevity). Future studies should investigate whether animal vocal communication systems demonstrating a fit to the Law of Brevity share functional constraints on call amplitude reduction; or, similarly to human language, have limited needs for loud calling.

Associate Editor: A. Gardner
